# Predicting length of stay from an electronic patient record system: a primary total knee replacement example

**DOI:** 10.1186/1472-6947-14-26

**Published:** 2014-04-04

**Authors:** Evelene M Carter, Henry WW Potts

**Affiliations:** 1 Information Services Department, Oxford University Hospitals NHS Trust, Oxford, UK; 2 Institute of Epidemiology and Health Care, University College London, London, UK

**Keywords:** Length of stay, Regression analysis, Models, statistical, negative binomial, Total knee replacement, Computerized Medical Records, Hospital planning

## Abstract

**Background:**

To investigate whether factors can be identified that significantly affect hospital length of stay from those available in an electronic patient record system, using primary total knee replacements as an example. To investigate whether a model can be produced to predict the length of stay based on these factors to help resource planning and patient expectations on their length of stay.

**Methods:**

Data were extracted from the electronic patient record system for discharges from primary total knee operations from January 2007 to December 2011 (n = 2,130) at one UK hospital and analysed for their effect on length of stay using Mann-Whitney and Kruskal-Wallis tests for discrete data and Spearman’s correlation coefficient for continuous data. Models for predicting length of stay for primary total knee replacements were tested using the Poisson regression and the negative binomial modelling techniques.

**Results:**

Factors found to have a significant effect on length of stay were age, gender, consultant, discharge destination, deprivation and ethnicity. Applying a negative binomial model to these variables was successful. The model predicted the length of stay of those patients who stayed 4–6 days (~50% of admissions) with 75% accuracy within 2 days (model data). Overall, the model predicted the total days stayed over 5 years to be only 88 days more than actual, a 6.9% uplift (test data).

**Conclusions:**

Valuable information can be found about length of stay from the analysis of variables easily extracted from an electronic patient record system. Models can be successfully created to help improve resource planning and from which a simple decision support system can be produced to help patient expectation on their length of stay.

## Background

Length of stay (LoS) is an important metric for assessing the quality of care and planning capacity within a hospital. It is a key performance indicator for the Department of Health (DoH) in England, used to monitor hospital quality and manage patient expectation. The length of time patients spend in hospital beds is known to be a good representation of the amount of resource utilised, for example bed capacity, staffing and equipment. Average LoS is therefore published by operation and hospital on the Department of Health’s NHS Choices [1] website to help patients make choices on which hospital they attend.

Hospitals are constantly adapting to clinical and financial pressures driven by policy changes, including recent attention towards reducing LoS where differences between hospitals are shown to vary widely [2]. This continuous pressure for improvement requires hospitals to review their processes to become more cost efficient and more standardised to improve patient expectation. Gaining a better understanding of LoS provides an opportunity to reduce the time patients stay in hospital [3].

The DoH use a limited set of variables to produce a case-mix adjusted average LoS for benchmarking use for NHS Choices, i.e. age, gender, social deprivation and comorbidity. Research has shown that other variables can also affect LoS, for example discharge destination [4] and consultant [5].

Electronic patient record (EPR) systems are widely used within hospitals. Since these systems were introduced over 40 years ago, they have significantly improved with technological advances providing timely collection of hospital data with quick and easy access for analysis that was not possible from paper records. Many items of data are collected throughout the patient’s contact with a hospital, of which many items are submitted to the national database in England, Hospital Episode Statistics (HES), giving rich databases locally and nationally from which to extract.

These data are far from being used to their full potential. LoS has been widely researched across many different specialties and medical conditions within many countries [6-8] including the UK [4,9]. However the number of variables analysed has remained small in most research papers, concentrating on variables such as comparing medical interventions [10] or the before or after care of a patient [11]. No studies concentrate on using the wealth of data readily available from EPR systems.

Most studies on LoS have also not been subjected to well-designed modelling. LoS distribution is frequently positively skewed; it is therefore surprising to find that much LoS research analyses the mean LoS and where the models applied presume unskewed data. The studies either do not check for skew before embarking on their research, using linear regression [12,13], or the techniques used to counter the problem are of questionable value, e.g. Jonas et al [9] used logistic regression on a binomial split of their data and Smith et al. [5] applied a linear regression model to logged LoS that they describe as being “approximately” successful. In this study, the log transformation was not successful at producing even an approximate normal distribution.

This study uses EPR data to ascertain which variables significantly affect LoS and, using these, produces a model to predict future LoS, helping to improve resource planning and enabling the creation of a decision support tool to be used for improving patient expectations. Primary total knee operations have been used in this study as an example group that has a high profile in the area of improving length of stay nationally.

## Methods

### Subjects, setting and methods

This study has not used patient identifiable information and had no requirement to contact patients. The Research and Development Manager and the Caldicott Guardian at the Oxford University Hospitals NHS Trust approved use of the data for this study.

“A Caldicott Guardian is a senior person responsible for protecting the confidentiality of a patient and service-user information and enabling appropriate information-sharing. Each NHS organisation < *in England* > is required to have a Caldicott Guardian; this was mandated for the NHS by Health Service Circular” http://systems.hscic.gov.uk/data/ods/searchtools/caldicott

This study constitutes an audit or service evaluation and therefore no ethics approval was needed from the NHS Research Ethics Committee, as advised by the Trust’s Caldicott Guardian. This study was exempt from approval by the UCL Ethics Committee as constituting an audit, http://ethics.grad.ucl.ac.uk/exemptions.php.

NHS ordinary admissions for primary total knee operations were identified for patients discharged between January 2007 to December 2011 from the Nuffield Orthopaedic Centre (NOC) hospital (n = 2,130), a tertiary referral centre for specialist orthopaedic services with 105 elective beds in its musculoskeletal division. Primary total knee operations are categorised as Classification of Interventions and Procedures Codes (OPCS codes) W401, W411 and W421. Cancer patients were excluded using ICD Codes in line with NHS Choices guideline for measuring LoS. Only adult patients were included (patients >15 years old).

The following factors were analysed: seasonality of admission and discharge, admitted day of the week, gender, age, ethnicity, the distance a patient lives from the hospital (calculated as straight line distance from grid references), deprivation of where the patient lives (derived from the postcode using the Office of National Statistics Indices of Deprivation [14]), the country in which the patient resides, the commissioner who reimburses the cost of treatment, comorbidities, lead consultant (anonymised), discharge destination and discharge method.

Although the Health Resource Group (HRG) has been used in other models, for example the Kings Fund PARR Model [15], unfortunately the models have become redundant as the HRG model has been updated with major changes rendering these models unusable. Therefore, to protect the future of the modelling in this study, we suggest that diagnosis and procedure codes (ICD10 and OPCS codes) are used as an alternative as they have remained relatively consistent over the years.

Major comorbidities found in primary total knee replacements were classified using ICD10 diagnosis codes based on advice from local clinical coders. The following comorbidities were analysed: diabetes, renal failure, heart failure, retention of urine, difficulty swallowing, pulmonary embolism, and respiratory failure.

### Statistics

Initial descriptive and univariate analyses were carried out. Data were analysed on the significant effect on LoS using the following non-parametric statistical tests: the Mann-Whitney test where only two groups exist, Kruskall-Wallis test where more than two groups exist, and Spearman’s rank correlation coefficient where the data was continuous. A p-value ≤ 0.05 was considered to be significant.

**Figure 1:**
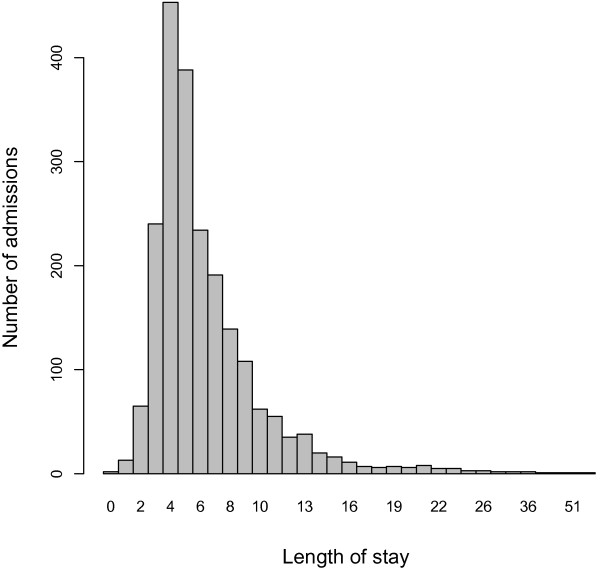
LoS distribution - primary total knees.

Length of stay is naturally a skewed distribution in most cohorts of patients, as shown in Figure 1 for this cohort.

Two modelling techniques were used, both appropriate for skewed data: Poisson and negative binomial regression. The negative binomial regression model is a common method used where the Poisson regression model does not explain all the variance. Both of these regression modelling techniques have had limited use in research of other specialties [16], however, no papers analysing the LoS of primary total knee replacements using these methods were found.

The data was randomly split into 90% of admissions to derive the model and the remaining 10% of admissions to test the model’s application. Residual plots were used to check model fit, including in terms of possible heteroscedasticity or data points with high leverage.

The analysis was carried out using R, a free package from the R Foundation for Statistical Computing [17].

## Results

### Descriptive and univariate analyses

The median LoS for primary total knee replacements (PTKs) was 5 days (the same as the national average), with a mean LoS of 6.4 days, showing positive skew.

There were no significant seasonal effects on LoS for PTKs, however there has been a significant reduction in the average (median) LoS in 2011 from 5 to 4 days (p <0.0001), suggesting a known program of reducing LoS for PTKs in the hospital was successful.

The day of the week a patient was admitted to the hospital proved to be significant (p <0.0001), with the median ranging between 4 and 9 days by day of the week. Patients admitted on a Tuesday and Wednesday stayed for 1 day longer (6 days) than those admitted on a Monday or Friday. It is known that discharges on a Saturday or Sunday were unlikely due to minimal clinical experience available at the weekend, which is likely to be the reason for this increased length of stay. A Saturday admission had the lowest median LoS at 4 days. The shorter stay is due to simpler cases being admitted for their operation because of the previously mentioned reduced medical support at the weekend. So, although day of the week is significant, it is actually explaining how some of the operational processes affect LoS, which it would not be possible to capture directly in an EPR system.

The average age of patients having a PTK operation was 70 years old (range 18 to 94), matching the profile of the national average (HES 2010–11 [18]). There was a U-shaped relationship with age whereby patients aged 60 stayed the shortest time and patients older or younger had increasingly longer LoS. A quadratic function seemed appropriate so Age^2^ was tested and the quadratic component was found to be significant (p <0.0001).

There were more women (59.8%) than men (40.2%) who were admitted for a PTK operation, with women staying one more day than men (p <0.0001).

There was up to 2 days significant difference in LoS depending on which consultant the patient is admitted under (p <0.0001), likely to be due to consultants’ individual specialties and case mix of patients.

Discharge destination contains a number of different responses with small numbers of admissions in most, so these were categorised into 3 simple groups for ease of modelling. This grouping was significant in predicting LoS (p <0.0001). The majority of patients are discharged home (94.6%), however those discharged to another destination which is not an NHS hospital provider stay for double the time (10 days) and those discharged to another NHS hospital provider stay for 8 days.

LoS significantly increases for those patients living in more deprived areas (p = 0.0097).

There is a high coverage of valid ethnicity data (92%). Only 2.4% of patients undergoing PTKs were of non-white ethnic origin but this group were found to have a significantly higher LoS by 1.5 days (p = 0.008).

The initial results of these significant factors are shown in Table 1.

**Table 1:** LoS analysis significant factor results

**Group**	**N**	**Proportion**	**Median**	**Mean**	**Median average deviation**	**Proportion %**	**Median deviation range**
<2011	1748	82.1%	5	6.6	1.48	82.1	3.5–6.5
2011	382	17.9%	4	5.7	1.48	17.9	2.5–5.5
Monday	449	21.1%	5	6.0	1.5	21.1	3.5–6.5
Tuesday	186	8.7%	6	7.4	3.0	8.7	3–9
Wednesday	356	16.7%	6	6.7	3.0	16.7	3–9
Thursday	394	18.5%	5.5	6.7	2.2	18.5	3.3–7.7
Friday	359	16.9%	5	6.2	1.5	16.9	3.5–6.5
Saturday	322	15.1%	4	5.2	1.5	15.1	2.5–5.5
Sunday	64	3.0%	9	10.7	4.4	3.0	4.6–13.4
Female	1274	59.8%	6	6.8	2.97	59.8	3–9
Male	856	40.2%	5	5.9	2.97	40.2	2 **-** 8
C58	274	12.9%	5	6.3	1.5	12.9	3.5–6.5
C38	256	12.0%	5	6.2	1.5	12.0	3.5–6.5
C49	246	11.5%	5	6.3	3.0	11.5	2–8
C46	211	9.9%	5	6.6	3.0	9.9	2–8
C42	200	9.4%	6	7.0	3.0	9.4	3–9
C59	179	8.4%	4	5.1	1.5	8.4	2.5–5.5
C26	171	8.0%	5	5.9	1.5	8.0	3.5–6.5
C62	156	7.3%	6	7.7	3.0	7.3	3–9
C64	148	6.9%	6	6.3	3.0	6.9	3–9
Other consultant	289	13.6%	5	6.9	2.97	13.6	2–8
NHS hospital provider	88	4.1%	8	9.8	7.4	4.1	0.6- 15.4
Other discharge destination	26	1.2%	10	11.7	5.2	1.2	4.8–15.2
Usual place of residence	2016	94.6%	5	6.2	1.5	94.6	3.5–6.5
White, declined and unknown	2074	97.6%	5	6.4	1.5	97.6	3.5–6.5
Other ethnicity	50	2.4%	6.5	7.6	3.7	2.4	2.8–10.2
**Factor**	**Mean**	**First Quartile**	**Median**	**Third Quartile**	**Median LoS at 40**	**Median LoS at 60**	**Median LoS at 80**
Age	70	64	71	77	7.5	5	6
**Factor**	**Mean**	**First Quartile**	**Median**	**Third Quartile**	**IMD at 3 Days LoS**	**IMD at 5 Days LoS**	**IMD at 7 Days LoS**
Indicies of deprivation	14720	7526	15353	22400	24368	24189	24011

The commissioning area within which the patient lived was not significant in predicting LoS for PTK operations (p = 0.20). Nor was the distance they lived from the hospital (p = 0.52).

Almost 100% of patients are discharged on clinical advice or with clinical consent (99.6% of admissions) but the data shows LoS is much longer for the handful of patients who die in the hospital. This paper does not attempt to predict whether a patient will die and therefore this variable is not used to model LoS.

Comorbidities were analysed based on ICD10 codes; however, the proportion diagnosed with these comorbidities was small. The comorbidity results were: diabetes (p = 0.21, 10% of cohort), renal failure (p = 0.077, 0.8% of cohort), heart failure (p = 0.52, <0.1% of cohort), retention of urine (p <0.001, 1.2% of cohort), difficulty in swallowing (p = 0.081, <0.1% of cohort) and pulmonary embolism (p = 0.038, 0.9% of cohort). Where the significant test suggested a good predictor of LoS, the cohort was too small where the criterion for a reliable significant test was set to be a minimum of 5% of the cohort per group. The remaining comorbidities were insignificant in predicting LoS. Respiratory failure was also analysed but no PTK admissions were diagnosed with this comorbidity.

### Modelling results

Two modelling methods were considered, the Poisson Regression Model (PRM) and the Negative Binomial Model (NBM). The PRM is a popular statistical modelling technique used for this type of skewed data and the outcome is relatively simple to explain. PRM has had limited use in research of other specialties [16], however, none of these analyse primary total knee replacements. The NBM is a similar model to the PRM and is a common method used where the PRM does not explain all the variance. No papers could be found that used the NBM to predict LoS in any specialty.

We fitted multiple regression models. We did this first for the PRM. We built the model by sequentially testing for the inclusion of each new, independent variable. If the variable’s addition was significant at the 5% level, it was retained in the model. We tested those variables that showed a significant relationship in the univariate tests, adding these in order from lowest to highest univariate p-value (see ‘order of modelling’ column in Table 2). The PRM fit the data moderately well, but showed evidence of over-dispersion, so we then explored the NBM. For comparison purposes and given all these variables appeared important (being significant in univariate tests and the PRM), we then included the same variables in the NBM as the PRM.

**Table 2:** Summary of univariately significant independent variables on LoS

**Variable**	**p-value**	**Test type**	**Test value**	**Order of modelling**
Admission year	<0.0001	Mann-Whitney	U = 104842283	1
Age at admission	<0.0001	Spearman’s	r = 0.26	2
Age^2^	<0.0001	Spearman's	r = 0.26	3
Gender	<0.0001	Mann-Whitney	U = 652862	4
Consultant	<0.0001	Kruskal-Wallis	*χ*^2^(9) = 75.76	5
Admission day of week	<0.0001	Kruskal-Wallis	*χ*^2^(6) = 146.15	6
Discharge destination	<0.0001	Kruskal-Wallis	*χ*^2^(2) = 35.37	7
IMD	0.01	Spearman’s	r = 0.06	8
Ethnicity	0.03	Mann-Whitney	U = 68095	9

**Figure 2:**
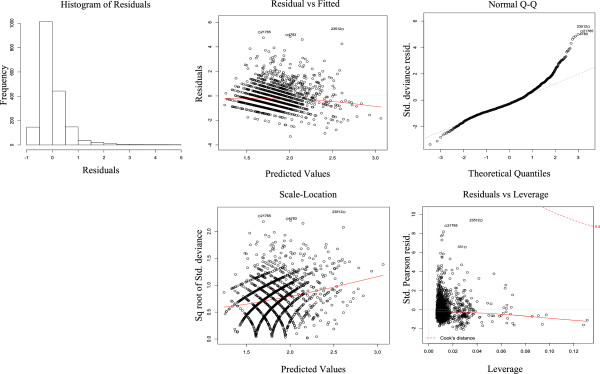
Negative binomial model residual plots.

**Figure 3:**
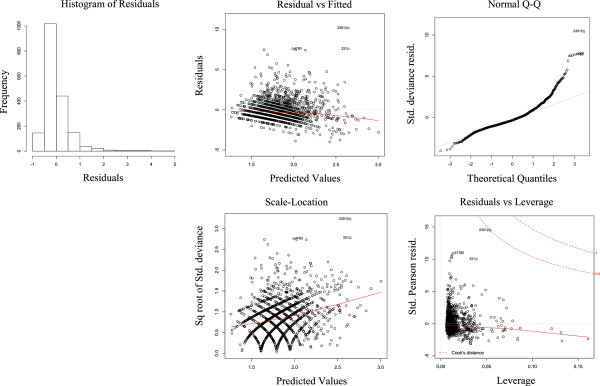
Poisson model residual plots.

The standardised deviance residuals of the NBM fit closer to a Normal distribution than the residuals of the PRM and there was stronger evidence that there were no overly influential points to the NBM as shown in Figures 2 and 3. The Akaike Information Criterion (AIC) is another measure to compare the quality of models [19]. The results of the AIC test applied to the NBM and PRM (Table 3) shows a large decrease in the NBM AIC compared to the PRM, showing that the NBM is the better model and that it is not plausible that the PRM is the best model. The model likelihood of 1 for the NBM also shows it is also very likely that the NBM is the correct model over the PRM.

**Table 3:** Akaike information criterion for the models

**Model**	**AIC**	**Diff AIC**	**Model likelihood**	**AIC weight**
Negative binomial	9170	0	1.0	1.0
Poisson	9668	498	0.0	0.0
	Total model Likelihood		1.0	

The NBM was therefore the model of choice where it showed less variance in the residuals and performed better when using the AIC. (Details of the PRM are available from the authors on request).

The formula structure for the predicted LoS is the exponential of all the coefficients multiplied by the variable value as shown in Equation 1 below.

Model Equation

(1) LoS=exp(Intercept+bx1*cfYear=2011+Age*cfAge+Age^2*cfAge^2+bx2*cf(Gender=Male)+bx3*cfCons=C38+etc.)

(where cf(*variable*) is the coefficient variable and b(x_n_) is a binomial variable with a value of 1 or 0)

The results are in the form of log-ratios, the Incident Rate Ratios (IRR). The results, as shown in Table 4, translate as shown in the following two examples:

• The IRR of a patient discharged to their usual place of residence is 0.72:1 i.e., this patient will stay only 72% of the time of a patient discharged to a NHS Hospital Provider.

• The IRR of a patient discharged to another discharge destination (i.e., not discharged home or to an NHS provider) is 1.11:1 i.e. the patient will stay 11% longer than a patient discharged to a NHS Hospital Provider.

**Table 4:** Summary of coefficients and incident rate ratios (IRR)

	**Coefficient**	**Std. error**	**z value**	**Pr (>|z|)**	**Significance**	**IRR**
(Intercept)	3.51500	0.32630	10.772	<2e-16	***	33.62
Admission.Year.Group2011	-0.0999	0.03409	-2.930	0.00339	**	0.90
Age.at.Admission	-0.04659	0.00933	-4.996	0.00000	***	0.95
Age.Squared	0.00043	0.00007	6.160	0.00000	***	1.00
GenderMale	-0.13760	0.02525	-5.450	0.00000	***	0.87
Consultant.Pseudo.Code.PK.GroupC38	0.07489	0.05752	1.302	0.19292		1.08
Consultant.Pseudo.Code.PK.GroupC42	0.11630	0.05977	1.946	0.05164	.	1.12
Consultant.Pseudo.Code.PK.GroupC46	0.13800	0.06180	2.234	0.02551	*	1.15
Consultant.Pseudo.Code.PK.GroupC49	0.01046	0.05734	0.182	0.85523		1.01
Consultant.Pseudo.Code.PK.GroupC58	0.06061	0.05859	1.034	0.30093		1.06
Consultant.Pseudo.Code.PK.GroupC59	-0.07585	0.06385	-1.188	0.23481		0.93
Consultant.Pseudo.Code.PK.GroupC62	0.17290	0.06781	2.550	0.01077	*	1.19
Consultant.Pseudo.Code.PK.GroupC64	0.03859	0.06718	0.574	0.56574		1.04
Consultant.Pseudo.Code.PK.GroupOther consultant	0.12170	0.05685	2.142	0.03223	*	1.13
Admission.DayMonday	-0.01045	0.04263	-0.245	0.80631		0.99
Admission.DaySaturday	-0.16110	0.04504	-3.578	0.00035	***	0.85
Admission.DaySunday	0.45220	0.07280	6.211	0.00000	***	1.57
Admission.DayThursday	0.00404	0.04522	0.089	0.92877		1.00
Admission.DayTuesday	0.10370	0.05123	2.024	0.04300	*	1.11
Admission.DayWednesday	0.01796	0.04357	0.412	0.68018		1.02
Discharge.Destination.PK.GroupOther discharge dest	0.10560	0.11270	0.937	0.34855		1.11
Discharge.Destination.PK.GroupUsual place of resid	-0.32660	0.05522	-5.915	0.00000	***	0.72
Rank.of.IMD.Score	-0.000005	0.00000	-2.901	0.00372	**	1.00
Ethnicity.Common.GroupWhite, declined and unknown	**-**0.13000	0.07777	-1.671	0.09467	.	0.88

0 = ‘***’ : 0.001 = ‘**’ : 0.01 = ‘*’ 0.05 : ‘.’ = 0.1 : ‘ ’ = 1.

The residuals were analysed to understand the fit of the negative binomial model to the data. They were found to be skewed like a Poisson distribution as would be expected in a good negative binomial model, shown in Figure 2. The residuals plotted against the predicted LoS shows a random nature with no obvious trend, except a slight decrease in the negative residuals towards the higher predicted values. The residual analysis also shows no leverage points i.e. there are no admissions with a LoS that has a big influence on the model.

**Figure 4:**
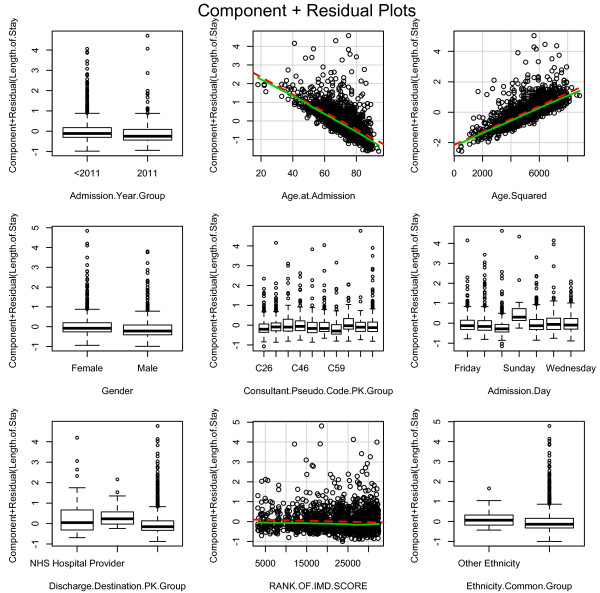
Negative binomial model residual plots by variable.

There is a large spread of residuals against most individual variables due to the skewed nature of the data, shown in Figure 4. The categorical variables show the residuals have an approximate mean of zero i.e. a good model fit. The IMD also shows a mean residual close to zero, whereas the trend seen in Age and Age^2^ shows the points are not randomly scattered nor is the mean consistent at zero, i.e. the model does not fully explain LoS with age.

The model was created using 90% of the data, “model data” (1,878 admissions), and tested on the remaining 10%, “test data” (252), to ensure the model did not over-fit the data. For the 11,805 total days the PTK patients stayed in hospital for (model data), the model has predicted 11,810 days; a difference of only 5 days over 5 years (see Table 5). Comparing this to the test data also shows very good results where only 88 more days were predicted than the actual 1,262 days, an overestimation of just 7% (see Table 6). The accuracy in predicting a patient’s stay is also impressive where, for those staying for 4 to 6 days, the model accurately predicted their stay, within 2 days, 74.7% of the time for the model data and 67.6% for the test data.

**Table 5:** Model results – model data

**LoS grouping**	**Proportion of admissions**	**Total number of actual days stayed**	**Total number of days difference (Model vs Actual)**	**% difference in total number of days**	**Predicting within 1 days accuracy**	**Predicting within 2 days accuracy**	**Predicting within 3 days accuracy**
4 to 6 days	50.8%	4469	1193	26.7%	41.1%	74.7%	91.4%
Shorter LoS	14.8%	729	780	107.0%	2.2%	30.4%	61.1%
Longer LoS	34.4%	6607	-1968	**-**29.8%	24.3%	42.1%	59.2%
**Total**	**100.0%**	**11805**	**5**	**0.0%**	**29.6%**	**56.9%**	**75.8%**

**Table 6:** Model results – test data

**LoS grouping**	**Proportion of admissions**	**Total number of actual days stayed**	**Total number of days difference (Model vs Actual)**	**% difference in total number of days**	**Predicting within 1 days accuracy**	**Predicting within 2 days accuracy**	**Predicting within 3 days accuracy**
4 to 6 days	50.9%	500	158	31.6%	32.4%	67.6%	88.6%
Shorter LoS	17.3%	95	110	115.6%	0.0%	30.6%	58.3%
Longer LoS	31.8%	667	-180	-27.0%	25.4%	52.2%	68.7%
**Total**	**100.%**	**1262**	**88**	**6.9%**	**24.5%**	**56.3%**	**76.9%**

However, when breaking the results down further, the model shows a few weaknesses. The results are shown in 3 groups, those staying 4 to 6 days and those staying for a shorter (<4 days) or longer (>6 days) length of time. For example, for those staying 4 to 6 days, the model predicts 26.7% more days for the model data and 31.6% more days in the test data. Also, the prediction accuracy within 2 days is not as accurate for those staying less than 4 days or more than 6, 30.4% and 42.1% respectively, for the model data, although the test data shows better prediction for longer stayers at 52.2%. Overall, the model predicts LoS well.

A worked example is provided to explain the model and the effects of each variable on LoS. The example patient, whom we refer to as Mrs Everett, is defined based on average characteristics of a patient undergoing a primary total knee operation:

• a 70 year old female

• admitted under consultant C58

• admitted on a Monday

• discharged to her normal place of residence

• with an IMD Rank of 24871.5

• of white ethnic origin

The formula used to calculate the LoS of the average patient is as follows:

$$\begin{array}{c} \mathrm{LoS}=\exp^{\left(3.515+\left(-0.0999\right)+\left(-0.04659*70\right)+\left(0.0004*70^{2}\right)+\left(0\right)+\left(0.06061\right)+\left(-0.01045\right)+\left(-0.3266\right)+\left(2.48715*-0.000005\right)+\left(-0.13\right)\right)}=5.5\mathrm{days} \end{array}$$

Any patient admitted in the future will have a coefficient of 1 for the admission year variable where the LoS for 2011 admissions is 90% of the LoS in previous years. Mrs Everett is admitted after 2011 so her LoS is 0.4 days shorter than if she had been admitted prior to 2011, a 6.1 days stay.

Age has two coefficients, one for Age and one for Age^2^. As previously discussed, age has a quadratic relationship with LoS. Table 7 shows the LoS per decade of age keeping all other aspects of Mrs Everett’s admission the same. The quadratic nature of age in the model can easily be seen.

**Table 7:** LoS for the average patient by age bands

**Age**	**LoS for the average patient**
20	8.4
30	6.5
40	5.5
50	5.1
60	5.1
70	5.5
80	6.6
90	8.5

A man will only stay 87% of the time of a woman. If a man was admitted with the same admission and discharge attributes of Mrs Everett his LoS reduces by 0.7 days to 4.8 days stay.

A patient admitted to consultant C58, for example, will stay for 6% longer than if they are admitted to consultant C26. For Mrs Everett, a consultant change from C58 to C26 would reduce her LoS by 0.3 days.

A patient admitted on a Monday will have a similar LoS as a patient admitted on a Friday (99% of that of a patient who is admitted on Friday). For Mrs Everett, the model estimates that changing the admission day to Friday will increase her stay to 5.6 days from 5.5 days, 0.1 days longer. Although not a noticeable difference, being admitted on other days has a much larger impact. For example, if the same patient is admitted on a Sunday the length of stay is predicted to be 8.8 days, 3.3 days longer.

A patient who is discharged to their usual place of residence will stay 72% of the duration of a patient who is discharged to another NHS hospital provider. Mrs Everett will stay 7.7 days if she is discharged to another NHS hospital provider, an extra 2.2 days.

The IMD rank ranges from 1 to ~35,000, therefore a patient who lives in an area that is only one rank lower on the derivation scale will see a negligible effect on their LoS, which can be seen in the model where the IRR for the IMD is very close to 1 (0.99999). However, a patient living in an area that is much lower in rank will result in a larger effect on LoS. For example, if Mrs Everett lived in an area with an IMD rank of 10,000 (a higher deprivation than 24,871.5), her LoS is estimated to be 6.0 days, 0.5 days longer.

A patient of white ethnicity will stay for 88% of the time of a patient of any other ethnicity. A patient from another ethnic group than Mrs Everett would stay 6.3 days, an extra 0.8 days than her.

## Discussion and conclusion

Patients, hospitals and national health services will benefit from an advanced understanding of factors that affect LoS because it is a good proxy for utilised resources. This study has provided an innovative model for predicting LoS enabling better planning of resources and has also been applied as a decision support tool to predict an individual patient’s LoS.

Research has contributed to this area but with questionable techniques using few variables and none utilising the wealth of data from an EPR system. This study complements and enhances current research by proving a number of variables extracted from an EPR system effect LoS and that these variables used in combination can produce a good model to predict LoS. The model fits the data well considering no clinical factors were included, and particularly well when analysing total number of days stayed, <1% error on the model data and 7% error on the test data. However, it should be noted that the model does not perform as well for short LoS.

We used Poisson and negative binomial regression models (the Poisson model can be considered a special case of the negative binomial model). As with all multiple regression modelling, the choice of what variables to include requires careful consideration. We developed our models using a forward-step iterative process that relies on the analyst to choose which variables to test in the model at each stage. As one variable is chosen to stay in the model, the next variable is tested based on the inclusion of those variables in the previous steps. Therefore the order chosen may not be the optimum. The variables were added by considering the order of best significance when tested as independent variables in univariate tests. All variables were assumed to be independent from each other.

The following factors were found to significantly affect LoS based on primary total knee replacements: year of admission, age, gender, consultant, day of admission, discharge destination, deprivation and ethnicity.

The effect of the year the patient was admitted on LoS reflects some of the previously known organisational changes that took place in the hospital in 2011. Smith et al. [5] and Crawford et al. [21] also found year of admission to be a significant in their analysis of LoS predictors in primary total knee operations. There was no seasonal effect on LoS providing some justification that care is standard regardless of what time of year the patient is admitted. There is, however, an obvious drop in activity and LoS for weeks 50 to 53 of the year and week 1 (the Christmas and New Year period). Neither consultants nor patients wish to be in hospital over the festive period and therefore effort is made within the hospital to reduce activity over this period. There were only 50 admissions (less than 5% of admissions) in weeks 52, 53 and 1 over the 5 year period so no statistically relevant comparison could be drawn. If a patient is admitted over the Christmas period they are likely to stay less time than the model predicts, therefore this is noted within the decision support tool for clinicians to advise patients of this.

Age was expected to be a predictor of LoS as it was found to be significant in many other research papers, including those researching other operations [5,14,21,22]. However, the quadratic nature was not anticipated. The residual trend showed that the model including Age^2^, although a better predictor than age alone, still does not fully explain LoS with age. A cubic age function may improve the model. Further research is needed here.

The study found that more women undergo a total knee replacement than men and that they stay an extra day on average, also found in other LoS research [9,22]. This could be explained by the difference in the perceived caring roles, as found by Barker et al. [23], where men possibly go home earlier because they have someone to look after them after being discharged.

Analysis at a consultant level was sensitively considered for this project. Differences in LoS between consultants are likely to be “due to chance alone” or “a quirk of case mix”, as suggested by Tavare et al. [24], rather than a measure of an individual’s performance. Transparency of the data should only be provided where context is also supplied. As this project does not attempt to detail the reasons behind this variance, to protect a competent consultant’s reputation, the decision was made to anonymise the consultants by allocating random pseudo codes. Further investigation is advised where a difference that is found in LoS between consultants is not expected based on their case mix. Smith et al. [5] also show consultants to be a predictive factor of LoS.

LoS by day of admission varied quite significantly, also explaining the organisational structure on discharge rules at the weekend, but does follow findings from Smith et al. [5]. Its addition in the model at the iteration stage after consultant proves that the variance seen in admission day is not fully explained by the regular operating days of the consultant, which had been a possibility. However, there may be a hidden effect of the regular operating days of an anaesthetist, which, unfortunately, cannot be determined by the data available from the EPR system. The building of a model on retrospective data produces a day of admission for all patients, however, when applying the model in the future approximately 40% of patients waiting for an operation do not have an admission date and therefore no admission day can be obtained. Proportional representation is recommended for each day in these cases. It can therefore be concluded that the closer the time period of prediction, the more patients will have a date for their admission i.e. the model will yield more accurate results.

There are known capacity issues in community hospitals within Oxfordshire (where the hospital is based) causing regular delays when attempting to transfer a patient there. It was therefore expected that a patient’s discharge destination would significantly affect LoS. Discharge destination was also found to significantly affect LoS in some other research [4,25], however, Zheng et al. [12] did not find discharge destination to be significant suggesting transfers were not an issue to community hospitals in that specific location. Although discharge destination is available retrospectively, this information is not collected within the EPR system prior to admission, even though often it is known. Therefore implementing the model as a future resource predictor directly from EPR will require proportional representation until such time it is collected, however, it can be used in the decision support tool when it is known where the patient will be discharged.

Deprivation shows a significant difference in LoS, even though the cohort of patients in this study live in less deprived areas. Cookson et al. [26] found a similar trend in hip replacement operations, although they found deprivation was not significant to predicting LoS. Where the patient has a missing or invalid postcode or a postcode outside of England, the average deprivation should be used in the model.

There was a high coverage of ethnicity recorded (92%), even though some research has claimed a lack of willingness of the patients to give it [27]. The result of a significant difference in LoS between white ethnic origin and other ethnic origins should be concluded on with caution. It does not imply that the hospital is discriminating against non-white ethnic groups, rather that there is likely to be a hidden correlation. For example, the difference in LoS may be associated with certain medical conditions found in different ethnic populations, or it could be due to the high tendency of people with non-white ethnic backgrounds to be living in more deprived areas with poorer health. Ethnic grouping has also been proven to have a significant effect on LoS in other in knee replacement research [20,28].

There was an expectation that patients travelling from longer distances to the hospital would stay longer, but this was not the case. This proves that patients are not being brought into hospital earlier for the convenience of the patient and therefore not blocking any beds. The exception to this is Maltese patients who were too few to model against, but it is known they are complex cases due to the hospitals contract with them. Although Maltese patients were not shown to significantly affect LoS, and is therefore not included in the model, an additional caveat has been added to the decision support tool to improve patient expectations.

Comorbidities did not have a significant effect on LoS, possibly due to the small proportion of patients who were diagnosed with those individual comorbidities. A wider cohort of patients would produce more information and therefore may show a significantly longer stay. Other research has shown diabetes, for example, significantly affects LoS [20], however, this paper analysed spinal operations where comorbidities may have a more serious effect on recovery time than knee operations. It is possible that other individual comorbidities, which have not been included in this project, have a significant effect on LoS. The Charlson score of co-morbidity could be used in further analysis as it is utilised by NHS Choices LoS prediction and within research [29]. However, it should be used with caution where a national update of this score could render a model redundant.

Admission type has been found to predict LoS in some research [4,25] and therefore could potentially improve the model. However, due the low quality of this data, a better understanding and an improvement in its collection in the EPR system is required.

**Figure 5:**
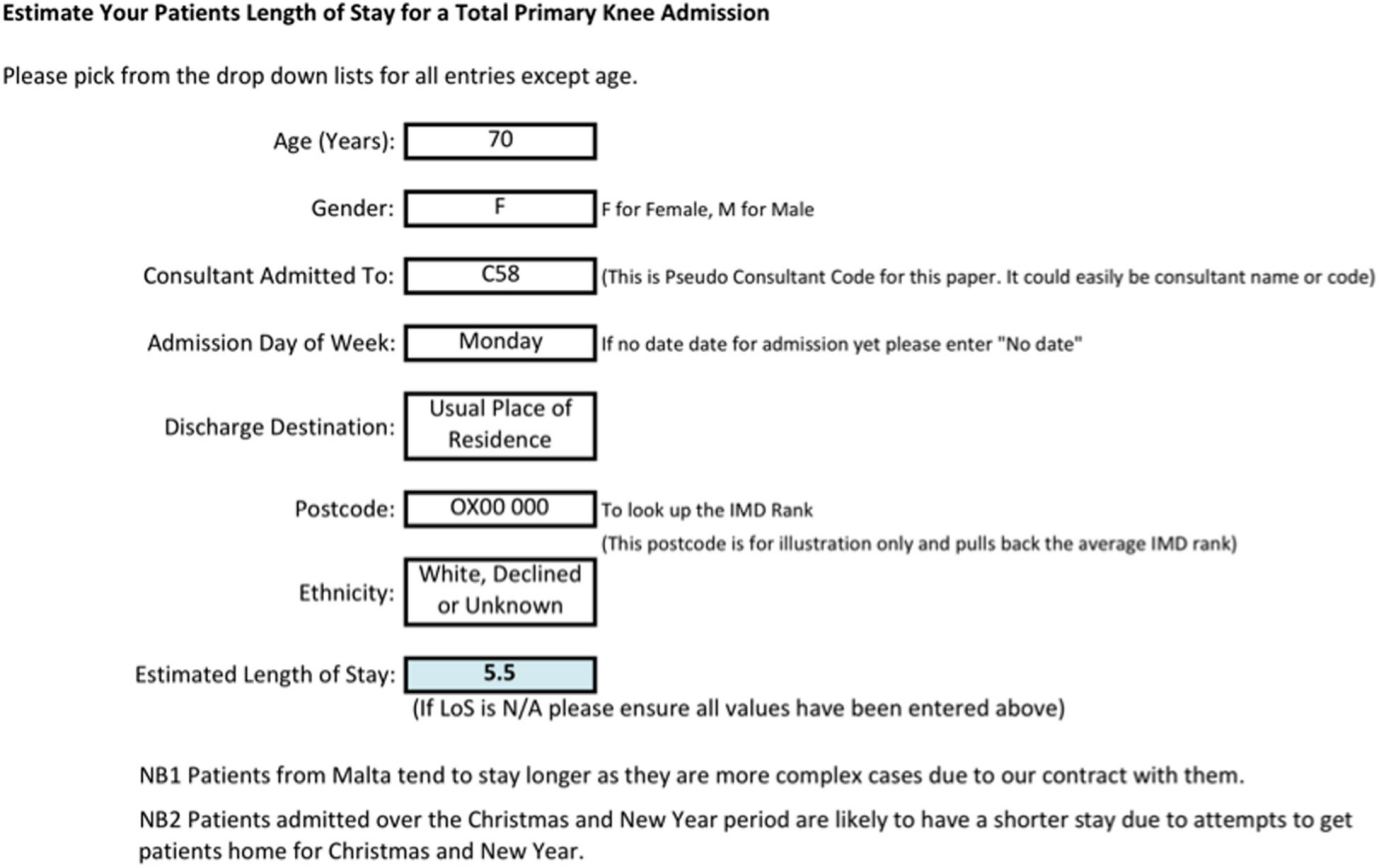
A screenshot of the decision support form for estimating a patients LoS.

This model can be applied to PTK patients admitted to any hospital in any country for resource planning and individual patient expectation of their LoS. The model can be easily implemented within this hospitals’ data warehouse and the decision support tool can be easily implemented (created in Excel). Figure 5 shows the decision support tool available to use at consultation before admission where the average patient, Mrs Everett, has been used in the example. Implementation of the model as a decision support system, to replace the decision support tool, could be carried out within the EPR system. However, the processes to implement this will be time consuming and this time lag to implementation would additionally affect any model updates that may be required in the future.

The more general modelling technique of negative binomial regression used in this study can be used for any type of cohort, i.e. for any type of operation within any hospital within any country where different patient characteristics, clinical pathways and organisational structures may be present. This modelling technique can also be used for predicting other key performance indicators, not just LoS, where skew is seen in the data. This flexibility is also enhanced where the statistical package used, R, is free and open source so the statistical methodology used in this study can be implemented by any hospital any where in the world for free, providing a major advantage on costly statistical software.

The model could be improved if other data sources were used in combination with this EPR data. Internally, for example, the hospitals separate theatre database could be used to calculate operation time as an operation complexity indicator. Clinically, other specialist databases could be linked in, for example the regional arthroplasty database where some research has found the stair score to have a significant effect on LoS [5], or the ASA score which was found to be significant in other research [9,21].

Valuable information can be found about length of stay from the analysis of variables easily extracted from an electronic patient record system. Models can be successfully created to help resource planning and from which simple decision support systems can be produced to help patient expectation. It is highly recommended that these statistical techniques are implemented to improve the future planning of national health services around the world.

## Competing interests

The authors declare no competing interests.

## Authors’ contributions

EC conceived the design of the study and carried out the design, implementation and analysis, including data collection, data analysis and statistical modelling. HP provided guidance throughout the project providing extensive feedback. Both authors read and approved the final manuscript.

## Pre-publication history

The pre-publication history for this paper can be accessed here:

http://www.biomedcentral.com/1472-6947/14/26/prepub
